# Photocatalytic Materials and Photocatalytic Reactions

**DOI:** 10.3390/molecules30020269

**Published:** 2025-01-11

**Authors:** Sugang Meng

**Affiliations:** 1 Key Laboratory of Green and Precise Synthetic Chemistry and Applications, Ministry of Education, College of Chemistry and Materials Science, Huaibei Normal University, Huaibei 235000, China; mengsugang@126.com; 2 Anhui Provincial Key Laboratory of Synthetic Chemistry and Applications, College of Chemistry and Materials Science, Huaibei Normal University, Huaibei 235000, China

## 1. Introduction

**Figure 1:**
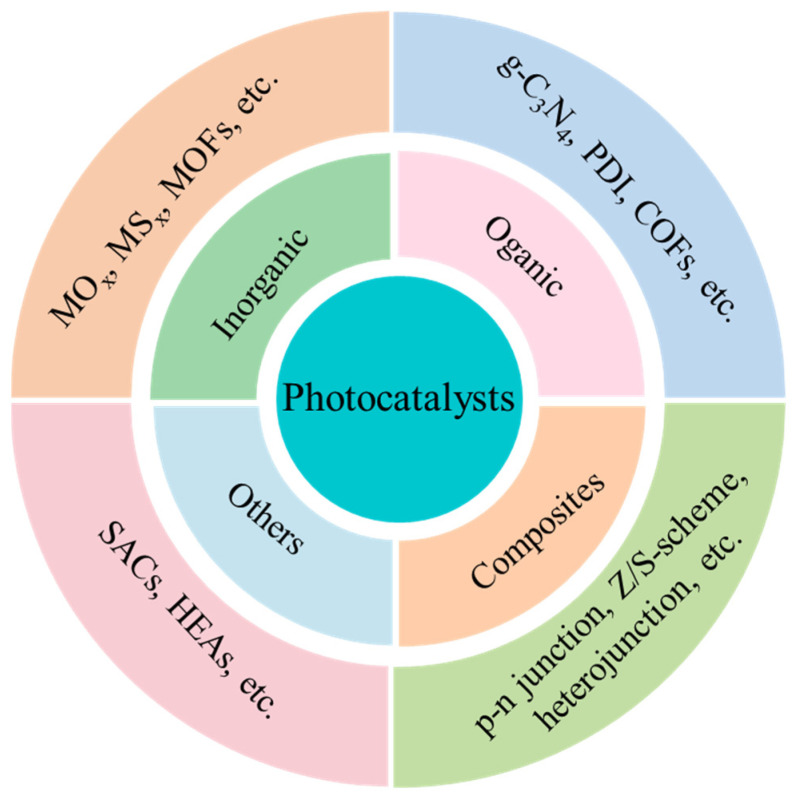
The types of materials explored for use as potential photocatalysts.

**Figure 2:**
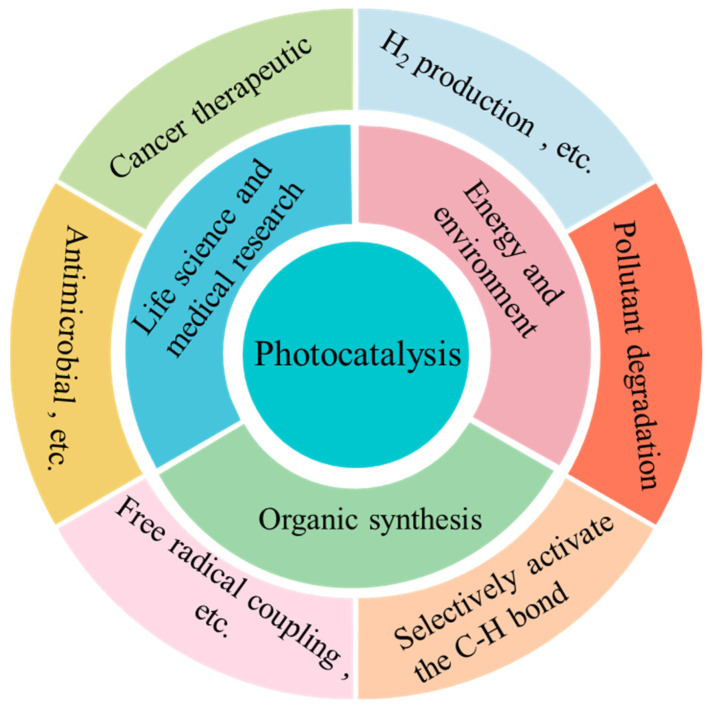
Various photocatalytic reactions.

This Special Issue, titled “Photocatalytic Materials and Photocatalytic Reactions”, focuses on designing advanced photocatalysts, understanding their structure-dependent properties, and seeking to exploit them in the fields of energy conversion, pollutant degradation, artificial photosynthesis, organic synthesis, etc. As early as 1912, in the age of coal, Giacomo Ciamician [1] proposed the theory that photochemistry could be a potential and promising strategy to realize the harmonious development of human society and nature. However, the pioneering work on photosynthesis was a study on photo-electrochemically water splitting, published in 1972 by Fujishima and Honda [2]. Throughout the next few years, photocatalysis was mainly studied in regard to the degradation of toxic compounds. In recent decades, photocatalysis has attracted extensive and ongoing attention, because it exhibits great potential for applications in artificial photosynthesis, including H_2_ production and CO_2_ reduction, organic synthesis, pollutant degradation, N_2_ fixation, precious metal recovery, H_2_O_2_ photosynthesis, life science and medical research, space exploration, and other related fields (Figure 1) [3,4,5,6,7,8,9]. Meanwhile, photocatalysts have evolved from inorganic substances to new nanomaterials such as graphitic carbon nitride (g-C_3_N_4_), polymers, piezoelectric materials, ferroelectric materials, metal–organic frameworks (MOFs), covalent organic frameworks (COFs), single-atom catalysts (SACs), high-entropy alloys (HEAs), supramolecules, superlattices, topological insulators, localized surface plasmon resonance (LSPR), and diverse composite materials/heterojunctions, among other things (Figure 2) [6,7,8,9,10,11,12,13,14,15,16].

**Figure 3:**
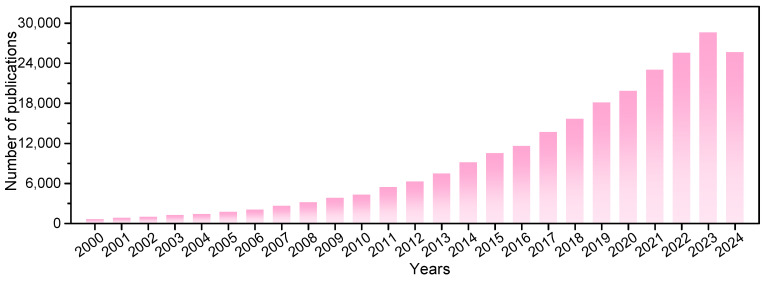
The number of annual journal publications that covered “photocatalysts*” as a subject prior to 9 December 2024, as recorded in the Web of Science database.

Our search results indicated a recent boom in photocatalysis research (Figure 3). About 243,000 studies on this topic have been published in the last 25 years. A total of 146 research areas and 212 countries/regions are involved. Photocatalysis is notable because it is driven by inexhaustible solar energy under mild reaction conditions. The design of advanced photocatalytic materials with good performance and the exploration of green photocatalytic reactions with carbon neutrality are significant for sustainability.

This Special Issue contains 23 original research articles related to photocatalytic materials, including metal oxides, metal sulfides, metal nitrides, metallo-organic compounds, g-C_3_N_4_, clusters, LSPR, and heterojunction/composite materials. Their applications included H_2_ production, CO_2_ reduction, organic synthesis, environmental remediation, disinfection, toxicity, and dual-function photoredox reactions.

## 2. An Overview of the Published Articles

In the Special Issue’s first contribution, Li et al. synthesized a new magnetic nanocomposite, Ag_2_O/Fe_3_O_4_, to achieve the photocatalytic degradation of methyl orange (MO) under visible light irradiation. They observed that 99.5% of MO could be degraded by the Ag_2_O/Fe_3_O_4_ (10%) photocatalyst within 15 min. In addition, the designed Ag_2_O/Fe_3_O_4_ photocatalyst also exhibited broad applicability and stability. Their work demonstrated successful photocatalysis–Fenton coupling and overcame the challenges involved in a difficult catalyst recovery process and regarding low photocatalytic efficiency. 

Next, in the study by Wang et al. (Contribution 2), a multicomponent composite MoP/a-TiO_2_/Co-ZnIn_2_S_4_ was prepared through multiple hydrothermal processes. The effects of Co dopants, i.e., amorphous TiO_2_ and MoP, increased visible light absorption, improving the separation of photoexcited charge carriers via heterojunction and hydrogen production sites, respectively. Thus, the high efficiency of photocatalytic H_2_ production was realized on MoP/a-TiO_2_/Co-ZnIn_2_S_4_. Compared to the pristine ZnIn_2_S_4,_ the H_2_ production of MoP/a-TiO_2_/Co-ZnIn_2_S_4_ was enhanced by about three times.

Furthermore, in the research work by Meng et al. (Contribution 3), nanostructured polymeric carbon nitride (g-C_3_N_4_, PCN) was synthesized using a one-step thermal polymerization process with the assistance of hot water vapor. Vapor has a dual function used to prepare nanostructured PCN: besides being a green etching reagent, it can act as a gas bubble template. Moreover, reaction times, temperatures, mechanisms were also studied in the precursors of PCN. The H_2_ production of nanostructured PCN increased by about four times in contrast to bulk PCN. This study offers a new and versatile strategy for fabricating nanostructured g-C_3_N_4_ with high photocatalytic performance.

Wang et al. (Contribution 4) outlined a visible light-induced regioselective cascade and the sulfonylation–cyclization of a cascade of 1,5-dienes under mild conditions. An array of 3-sulfonylated pyrrolin-2-one derivatives was constructed through lower catalyst loading and achieved good to excellent yields at room temperature. Importantly, this protocol can be used in large-scale synthesis.

Meanwhile, in the research paper by Meng et al. (Contribution 5), a series of Zn_m_In_2_S_3+m_ photocatalysts (m = 1, 2, 3, 4 and 5) was prepared and applied in dual-function photoredox reactions: the selective oxidation of alcohols and the reduction of CO_2_ in one reaction system. In Zn_5_In_2_S_8_, Zn_4_In_2_S_7_, Zn_3_In_2_S_6_, Zn_2_In_2_S_5_, and ZnIn_2_S_4_, structures and properties was studied using experiments and theoretical calculation. The morphology, light absorption, and band structures were tuned by changing the Zn/In molar ratio. Moreover, the selectivity of gas products (H_2_ and CO) and liquid products (hydrobenzoin and benzaldehyde) could also be regulated.

In the research work by Chen et al. (Contribution 6), Ag/PW12/TiO_2_ composed of TiO_2_, polyoxometalates (POMs) [H_3_PW_12_O_40_] (PW12), and Ag nanoparticles was fabricated through successive electrospinning and photoreduction processes. Ag/PW12/TiO_2_ exhibited high degradation efficiencies for methyl orange (MO, 99.29%), enrofloxacin (ENR, 93.65%), and tetracycline (TC, 78.19%). Moreover, for TC degradation and TC concentration, the Ag/PW12/TiO_2_ dosage, the pH of the TC solution, and the intermediates and toxicities of the products were also investigated in detail.

Furthermore, in the study by Zhou et al. (Contribution 7), the Bi_2_S_3_-ZnO/CA film was fabricated by assembling Bi_2_S_3_, ZnO, and cellulose acetate (CA). In the Bi_2_S_3_-ZnO/CA film, the addition of Bi_2_S_3_ improved cavity density and uniformity. On the other hand, the addition of ZnO enabled the formation of heterojunctions with Bi_2_S_3_ to promote the separation and migration of photogenerated electron–hole pairs, thus improving the photocatalytic activity and stability of Bi_2_S_3_. This is evidenced by the fact that the RhB (rhodamine B) degradation efficiencies of ZnO/CA, 4Bi2S3/CA, and 4Bi_2_S_3_-ZnO/CA were about 26.51%, 50.26%, and 90.2%, respectively.

Shahid et al. (Contribution 8) prepared a 2D I-FeWO_4_/GO composite photocatalyst by combining halogen-doped FeWO_4_ (I-FeWO_4_) and graphene oxide (GO). In the designed I-FeWO_4_/GO composite, GO acted as a supporter, halogen facilitated H_2_O_2_ production, and the interface of the I-FeWO_4_/GO heterostructure promoted charge separation and migration. Thus, the I-FeWO_4_ displayed good photocatalytic performance for the degradation of methylene blue (MB). Under sunlight irradiation for 120 min, 97.0% of MB could be degraded by the I-FeWO_4_.

Furthermore, Ai et al. (Contribution 9) fabricated 2D heterojunction g-C_3_N_4_@CdS using a water bath method. Interestingly, tiny CdS nanorods were grown in situ in the gaps between the 2D g-C_3_N_4_ nanosheets. Due to the band-band transfer mechanism of the g-C_3_N_4_@CdS photocatalyst, the charge carriers could be separated efficiently, and thus excellent H_2_ production with the assistance of visible light and lactic acid was observed. The H2 production rate of G-CdS-3 could reach up to 1611.4 μmol·g^−1^·h^−1^, which was about 10 times that of CdS and 76 times that of g-C_3_N_4_.

In the research work by Wang et al. (Contribution 10), a 2D–3D hybrid junction In_2_S_3_/CdS/N-rGO was synthesized using a one-step pyrolysis method. The rational 2D–3D In_2_S_3_/CdS/N-rGO hybrid junctions not only provided more active sites but also formed multiple tight interfaces, which facilitated charge separation and migration. Thus, a high H_2_ evolution rate (10.9 mmol·g^−1^·h^−1^) could be obtained with the assistance of visible light and Na_2_S/Na_2_SO_3_ aqueous solution.

To remove high levels of toxic 4-nitrophenol (4-NP), Ma et al. (Contribution 11) designed the alkaline earth metal ion-doped photocatalyst Ca^2+^-doped AgInS_2_. The charge recombination and inactive production of superoxide radicals over AgInS_2_ could be improved by doping Ca^2+^. Thus, 63.2% of 4-NP was degraded under visible light for 120 min. In addition, capturing tests demonstrated that photoexcited holes and hydroxyl radicals were the main active species.

In the research work by Lu et al. (Contribution 12), cobalt phosphate (Co-Pi) was developed to modify ZnIn_2_S_4_ (ZnIn_2_S_4_/Co-Pi), aiming to suppress its charge recombination. Due to the transfer of photoexcited holes, ZnIn_2_S_4_/Co-Pi exhibited sustainable H_2_ production with assistance of visible light and triethanolamine (TEOA). Specifically, 3593 μmol·g^−1^·h^−1^ of H_2_ production was reached using ZnIn_2_S_4_/5%Co-Pi.

To remove hexavalent chromium, Guo et al. (Contribution 13) prepared a 2D g-C_3_N_4_/MoS_2_ nanocomposite using an ultrasonic method. Due to the Z-scheme transfer mechanism, photoexcited charges could not only be separated efficiently at the g-C_3_N_4_/MoS_2_ heterojunction but they also retained strong redox abilities. Thus, hexavalent chromium could be removed with high photocatalytic efficiency whenever it was exposed to UV light, visible light, or sunlight.

Furthermore, Garcia et al. (Contribution 14) fabricated a PdIn/TiO_2_ hybrid photoelectrocatalyst for wastewater treatment with simultaneously clean energy production. In the reaction system, on the one hand, paracetamol was degraded by oxidation at the photoanode; on the other hand, hydrogen was produced through reduction at the photocathode. Thus, a dual-function redox reaction system was established.

To use sunlight for CO_2_ reduction, Wang et al. (Contribution 15) designed TiO_2_/CuPc heterojunctions by combining TiO_2_ microspheres and copper phthalocyanines (CuPc). Benefiting from the heterojunction effect and the additional light-absorbing properties of TiO_2_ and CuPc, a good reduction rate in 32.4 μmol·g^−1^·h^−1^ of CO_2_ was achieved with TiO_2_/CuPc at about 3.7 times that of the pristine TiO_2_.

Unlike the experiments above, Gao et al. (Contribution 16) utilized the extended broken symmetry (EBS) method to investigate the low-lying spin states of the [Fe_3_S_4_] cluster, which is important for photosynthetic H_2_O splitting. The results indicated that the EBS results matched well with the experimental data. The weaknesses of the BS method could be compensated for through the developed EBS method.

Meanwhile, Tang et al. (Contribution 17) investigated the Sc_2_CX_2_/Sc_2_CY_2_ (X, Y = F, Cl, Br) Janus heterojunction for photocatalysis and photovoltaics using first-principles calculations. The calculated results indicate that these Janus heterojunctions possess type-II band structures and direct Z-scheme transfer mechanisms, which thus facilitates their application in photocatalysis and photovoltaics.

In their research paper, P. Ávila-Torres et al. (Contribution 18) prepared metal-yielded coordination compounds Cu-g-C_3_N_4_, Ni-g-C_3_N_4_, and Mn-g-C_3_N_4_. The structural properties of disinfected E. coli bacteria were investigated. The results indicate that the textural property is a key characteristic.

Liu et al. (Contribution 19) studied hydroxyl groups on the g-C_3_N_4_ (HCN) for O_2_ activation and pollutant degradation. The results show that hydroxyl groups can increase hydrophilicity and surface area, decrease interlayer distances, and promote the charge separation and transportation of the pristine g-C_3_N_4_, thus improving rhodamine B degradation.

Furthermore, A. Pavlatou et al. (Contribution 20) developed a new photocatalyst, namely magnesium oxide (MgO). MgO has a wide band gap and can produce hydroxyl radicals. It showed selectivity for rhodamine 6G and rhodamine B degradation under UV light. A total of 100% of rhodamine B could be degraded over MgO under UV light for 180 min.

In the research paper by Narkiewicz et al. (Contribution 21), triethylamine (TEA), diethylamine (DEA), and ethylenediamine (EDA) were used to modify TiO_2_. The effect of amines and temperature on the photocatalytic reduction of CO_2_ was investigated in detail. The results demonstrated that TEA-TiO_2_ treated in the microwave reactor exhibited the highest activity. 

Furthermore, in the research by Wang et al. (Contribution 22), the 2D GaN/g-C_3_N_4_ heterojunction was investigated using first-principle calculations. The calculated results indicate that the GaN/g-C_3_N_4_ heterojunction possesses type-II band structures, broad light absorption capabilities, and direct Z-scheme transfer mechanisms, and thus has potential applications in the field of photocatalysis.

In the final research paper, Gerken et al. (Contribution 23) investigated the interplay between photocatalytic growth and the chemical dissolution of gold structures on TiO_2_/ITO patterns as a novel approach to mimic axonal dynamic connections. By optimizing gold growth and dissolution parameters, we demonstrated the potential for the precise control of the formation and removal of conductive pathways. This work bridges photocatalytic materials research and bio-inspired system development, offering new insights into the application of photodeposition and chemical etching in dynamic, adaptive systems.

## 3. Conclusions

In the face of increasingly severe environmental problems and resource challenges, green and sustainable development has become a global consensus and a guide for action. Photocatalysis is one of the most promising strategies for addressing the severe issues facing the environment and energy production and has attracted extensive and ongoing attention due to its inexhaustible, green, safe and economically viable characteristics. However, there are still many challenges involved in the practical application of photocatalysis, such as quantum efficiency, stability and reusability, selectivity, output-to-input ratio, and scaling-up. Fortunately, as a result of decades of hard work, photocatalysis has progressed to a new stage. Various and ingenious photocatalytic materials and photocatalytic reactions have been developed. Photocatalytic materials include but are not limited to nonmetallic LSPR materials, MOFs, COFs, SACs, SCCs, HEAs, heterojunctions, and composite materials. Photocatalytic reactions are involved in the fields of environmental, life science, medical research, space exploration, agriculture and food, energy, etc. However, designing advanced photocatalytic materials with good performance and exploring green photocatalytic reactions with carbon neutrality will require further progress.

## References

[1] Ciamician G. (1912). The photochemistry of the future. Science.

[2] Fujishima A., Honda K. (1972). Electrochemical photolysis of water at a semiconductor electrode. Nature.

[3] Lei J., Zhou N., Sang S., Meng S., Low J., Li Y. (2024). Unraveling the roles of atomically-dispersed Au in boosting photocatalytic CO_2_ reduction and aryl alcohol oxidation. Chin. J. Catal..

[4] Che Y., Weng B., Li K., He Z., Chen S., Meng S. (2025). Chemically bonded nonmetallic LSPR S-scheme hollow heterostructure for boosting photocatalytic performance. Appl. Catal. B Environ. Energy.

[5] Candish L., Collins K.D., Cook G.C., Douglas J.J., Gómez-Suárez A., Jolit A., Keess S. (2021). Photocatalysis in the life science industry. Chem. Rev..

[6] Kumar P., Singh G., Guan X., Lee J., Bahadur R., Ramadass K., Kumar P., Kibria M.G., Vidyasagar D., Yi J. (2023). Multifunctional carbon nitride nanoarchitectures for catalysis. Chem. Soc. Rev..

[7] Dhakshinamoorthy A., Li Z., Yang S., Garcia H. (2024). Metal–organic framework heterojunctions for photocatalysis. Chem. Soc. Rev..

[8] Li X., Mitchell S., Fang Y., Li J., Perez-Ramirez J., Lu J. (2023). Advances in heterogeneous single-cluster catalysis. Nat. Rev. Chem..

[9] Ham R., Nielsen C.J., Pullen S., Reek J.N. (2023). Supramolecular coordination cages for artificial photosynthesis and synthetic photocatalysis. Chem. Rev..

[10] Sayed M., Yu J., Liu G., Jaroniec M. (2022). Non-noble plasmonic metal-based photocatalysts. Chem. Rev..

[11] Zhang H., Gao Y., Meng S., Wang Z., Wang P., Wang Z., Chen S., Weng B., Zheng Y.-M. (2024). Metal sulfide S-scheme homojunction for photocatalytic selective phenylcarbinol oxidation. Adv. Sci..

[12] Su B., Kong Y., Wang S., Zuo S., Lin W., Fang Y., Hou Y., Zhang G., Zhang H., Wang X. (2023). Hydroxyl-Bonded Ru on Metallic TiN Surface Catalyzing CO_2_ Reduction with H_2_O by Infrared Light. J. Am. Chem. Soc..

[13] Meng S., Chen C., Gu X., Wu H., Meng Q., Zhang J., Chen S., Fu X., Liu D., Lei W. (2021). Effcient photocatalytic H_2_ evolution, CO_2_ reduction and N_2_ fxation coupled with organic synthesis by cocatalyst and vacancies engineering. Appl. Catal. B Environ..

[14] Nishiyama H., Yamada T., Nakabayashi M., Maehara Y., Yamaguchi M., Kuromiya Y., Nagatsuma Y., Tokudome H., Akiyama S., Watanabe T. (2021). Photocatalytic solar hydrogen production from water on a 100-m^2^ scale. Nature.

[15] Yang J., Li L., Xiao C., Xie Y. (2023). Dual-Plasmon Resonance Coupling Promoting Directional Photosynthesis of Nitrate from Air. Angew. Chem. Int. Ed..

[16] Liu D., Xu H., Shen J., Wang X., Qu C., Lin H., Long J., Wang Y., Dai W., Fang Y. (2024). Decoupling H_2_ and O_2_ Release in Particulate Photocatalytic Overall Water Splitting Using a Reversible O_2_ Binder. Angew. Chem. Int. Ed..

